# Supplementary material for: Distinct ankyrin repeat subdomains control VAPYRIN locations and intracellular accommodation functions during arbuscular mycorrhizal symbiosis
Source: Nat Commun. 2022 Sep 5;13:5228. doi: 10.1038/s41467-022-32124-3 (PMC9445082; doi:10.1038/s41467-022-32124-3)
Supplement: Supplementary file 1 — Supplementary information [file 41467_2022_32124_MOESM1_ESM.pdf]

Supplementary Information

Supplementary Figures

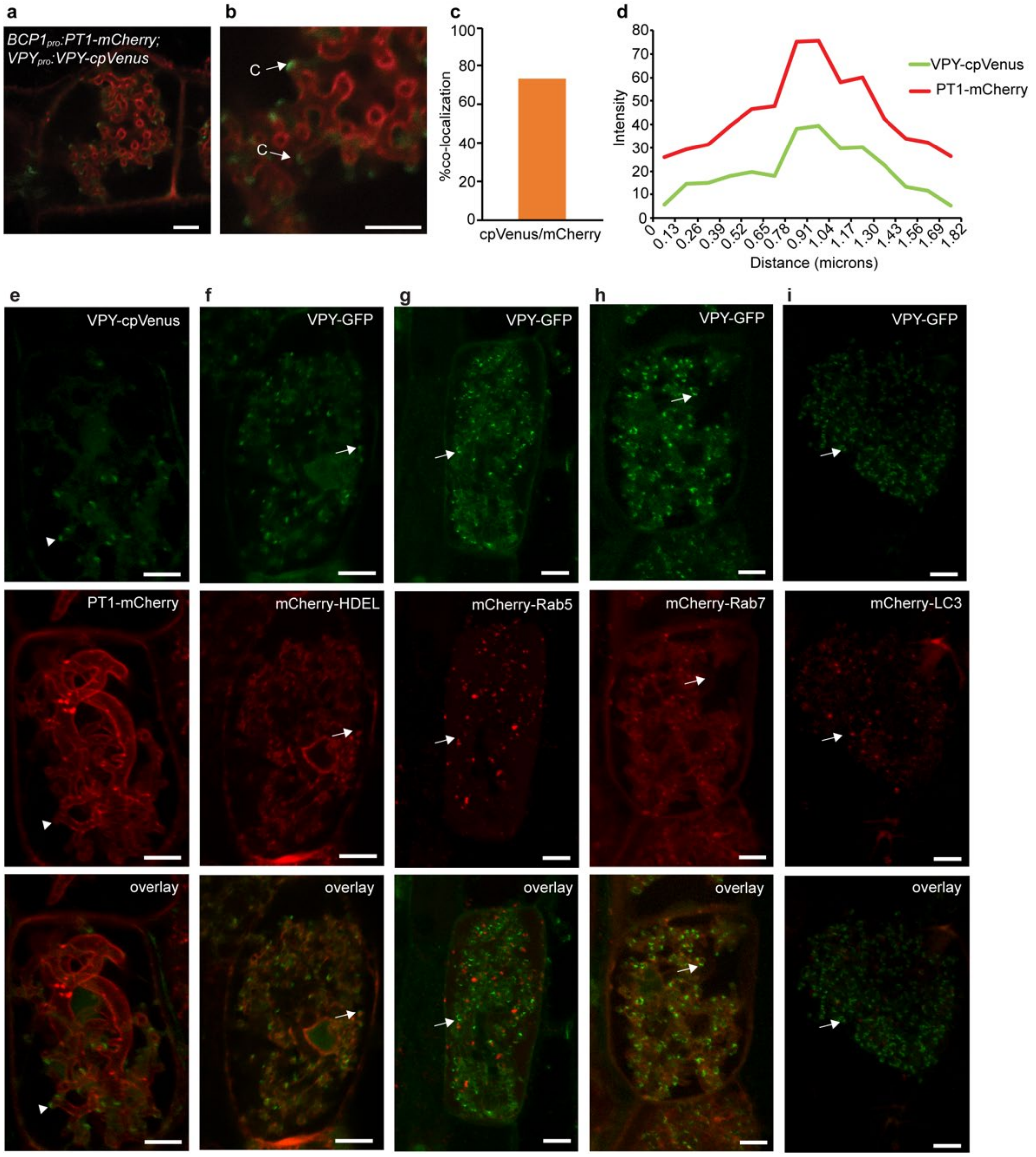

Supplementary Figure 1. A subset of the VPY punctate signals co-localize with the PAM and VPY does not co-localize with secretory pathway markers. (a) Live cell confocal image of arbuscule-containing cell, red is *$BCP1_{pro}$:PT1-mCherry* (PAM marker), green is *VPY_{pro}:VPY-cpVenus*. (b) Magnification of cell, two representative VPY signals co-localized with PT1-mCherry are indicated with arrows. (c) Percentage of co-localization between VPY-cpVenus punctate/crescent-shaped signals and PT1-mCherry, n=45 punctate/crescent-shaped signals from

two independent experiments and four biological replicates. (d) Representative linear transect showing overlapping intensity profile between VPY-cpVenus punctate/crescent shaped signals and PT1-mCherry PAM marker. (e) Co-expression of *VPY_{pro}:VPY-cpVenus* and periarbuscular membrane marker *$BCP1_{pro}$:PT1-mCherry* (n= 10 arbuscule-containing cells). (f) Co-expression of *VPY_{pro}:VPY-GFP* and ER marker *$BCP1_{pro}$:mCherry-HDEL* (n=11 arbuscule-containing cells). (g) Co-expression of *VPY_{pro}:VPY-GFP* and multi-vesicular body (MVB) marker *$BCP1_{pro}$:mCherry-Rab5* (n=8 arbuscule-containing cells). (h) Co-expression of *VPY_{pro}:VPY-GFP* and late endosome marker *$BCP1_{pro}$:mCherry-Rab7* (n=7 arbuscule-containing cells). (i) Co-expression of *VPY_{pro}:VPY-GFP* and autophagosome marker *$BCP1_{pro}$:mCherry-LC3* (n=7 arbuscule-containing cells). Arrowheads indicate representative VPY punctate signals that co-localize with mCherry marker, arrows indicate representative VPY punctate signals that do not co-localize with mCherry marker. Fluorescence images are projections of 10 optical sections on the z-axis taken at 0.5 μm intervals. Scale bar is 10 μm. For (d) – (i), representative images of a single root transformation experiment, with three root systems and three infection units per system imaged. The number of arbuscule-containing cells imaged for each marker is listed next to the marker.

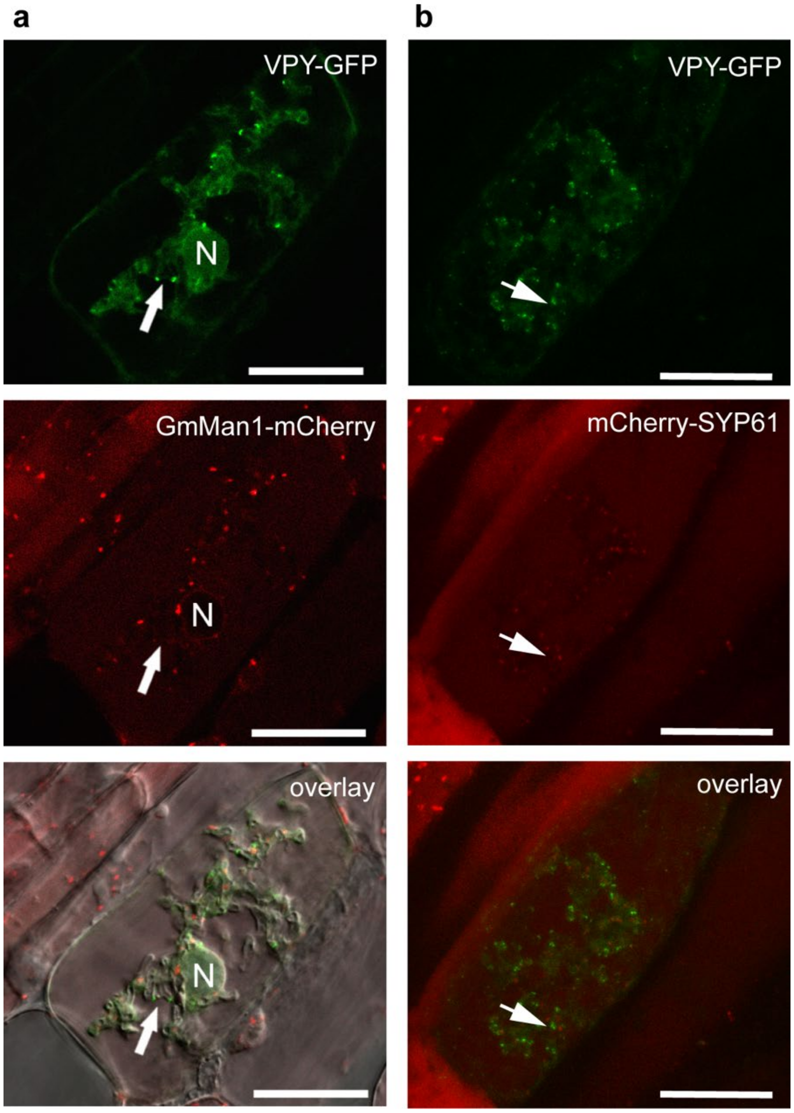

Supplementary Figure 2. VPY-GFP does not co-localize with the Golgi marker GmMan1 or TGN marker SYP61. (a) Co-expression of *VPY_{pro}:VPY-GFP* and Golgi marker *GmMan1-mCherry*. (b) Co-expression of *VPY_{pro}:VPY-GFP* and TGN marker *mCherry-SYP61.* Roots are colonized with *D. epigeae* Arrowheads indicate representative VPY punctate signals that do co-localize with mCherry marker. Scale bar is 20μm. The GmMan1 marker is comprised of the first 49 amino acids of GmMan1. Representative images of a single root transformation experiment, with n=5 arbuscule-containing cells for *GmMan1-mCherry*, and n=10 arbuscule-containing cells for *mCherry-SYP61.*

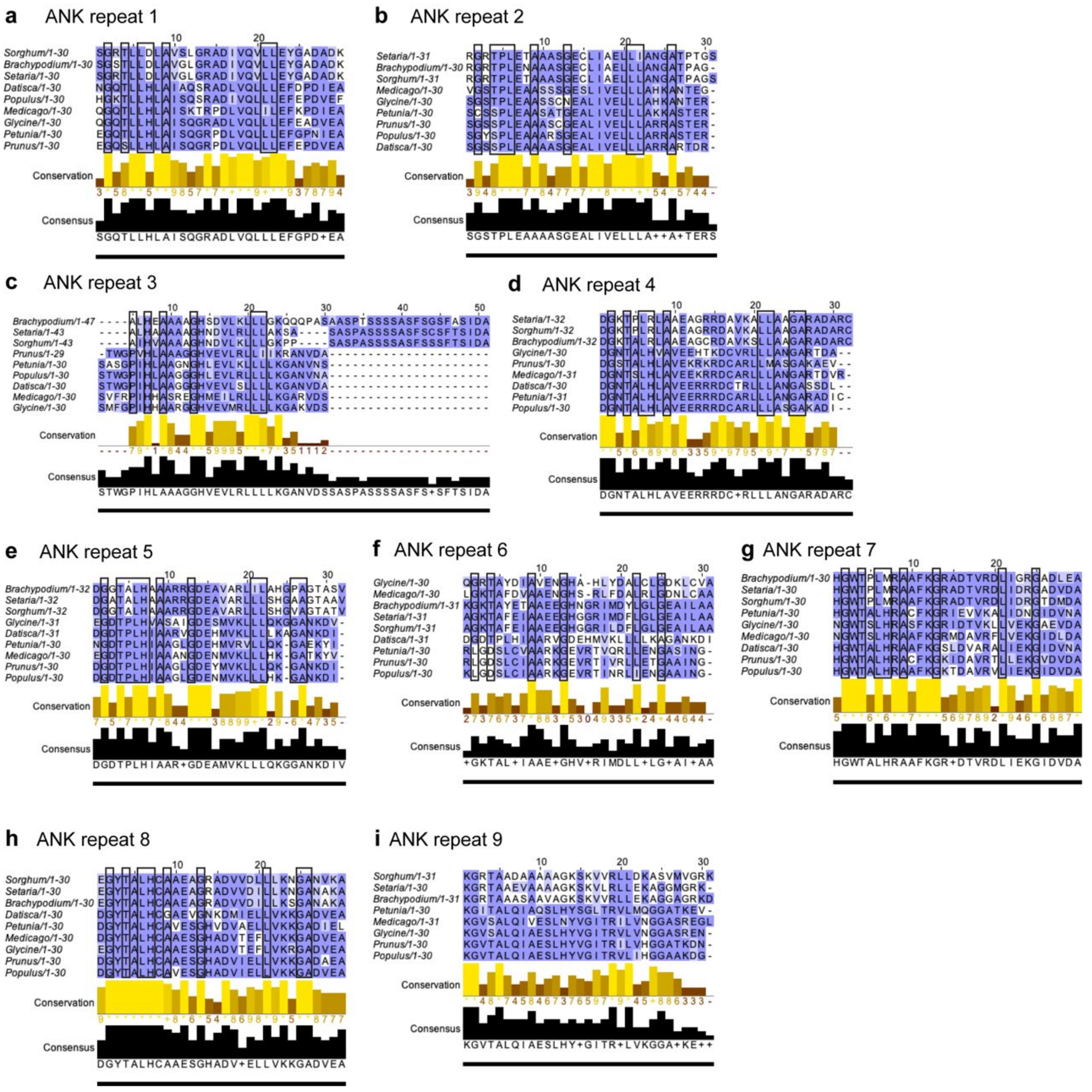

Supplementary Figure 3. Amino acid conservation of VPY ankyrin repeats among monocots and eudicots. Ankyrin repeats from several dicot and monocot species were aligned with MtVPY ANK repeats using Clustal Omega and visualized using JalView. Identical amino acids are in dark purple, similar amino acids are in light purple. Boxes indicate canonical ankyrin repeat amino acids. The amount of conservation is indicated with the yellow bars, and the consensus sequence is shown below the amino acid alignment. Yellow numbers indicate amount of conservation, with * indicating 100 % conservation. TXXH domains are present in repeats 1,4,5,7 and 8. The species used for the analysis are *Brachypodium distachyon* (GenBank ID: NC_016131), *Sorghum bicolor* (GenBank ID: NC_012870), *Setaria italica* (GenBank ID: NC_028458), *Petunia hybrida* (GenBank ID: FJ717417), *Medicago truncatula* (GenBank ID:

NC_016412), *Populus trichocarpa* (GenBank ID: NC_037297), *Glycine max* (GenBank ID: NC_038241), *Prunus persica* (GenBank ID: NC_034015), *Datisca glomerata* (GenBank ID: AGG82491).

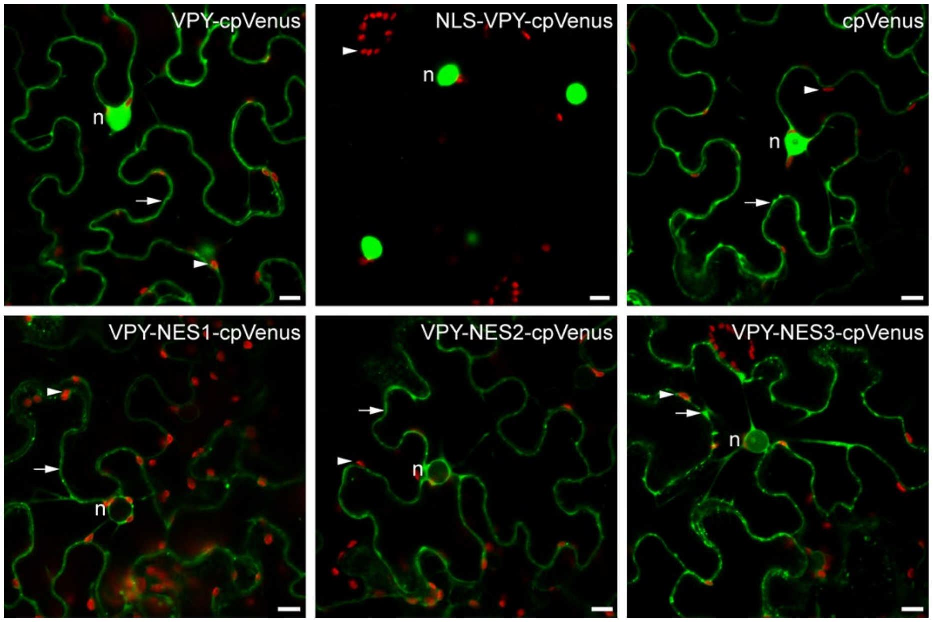

Supplementary Figure 4. Sub-cellular location of cpVenus-tagged VPY (n=23 cells), NLS-VPY (n=21 cells), VPY-NES1 (n=16 cells), VPY-NES2 (n=13 cells), VPY-NES3 (n=13 cells) and free cpVenus (n=10 cells) in pavement epidermal cells of *N. benthamiana* leaves. Fluorescent signal is detected either in nucleoplasm (*n*) or cytoplasm (*arrow*). *Arrowhead* - autofluorescence of chloroplasts. Confocal microscopy images. Scale bars, 10µm. Representative images of two independent infiltrations.

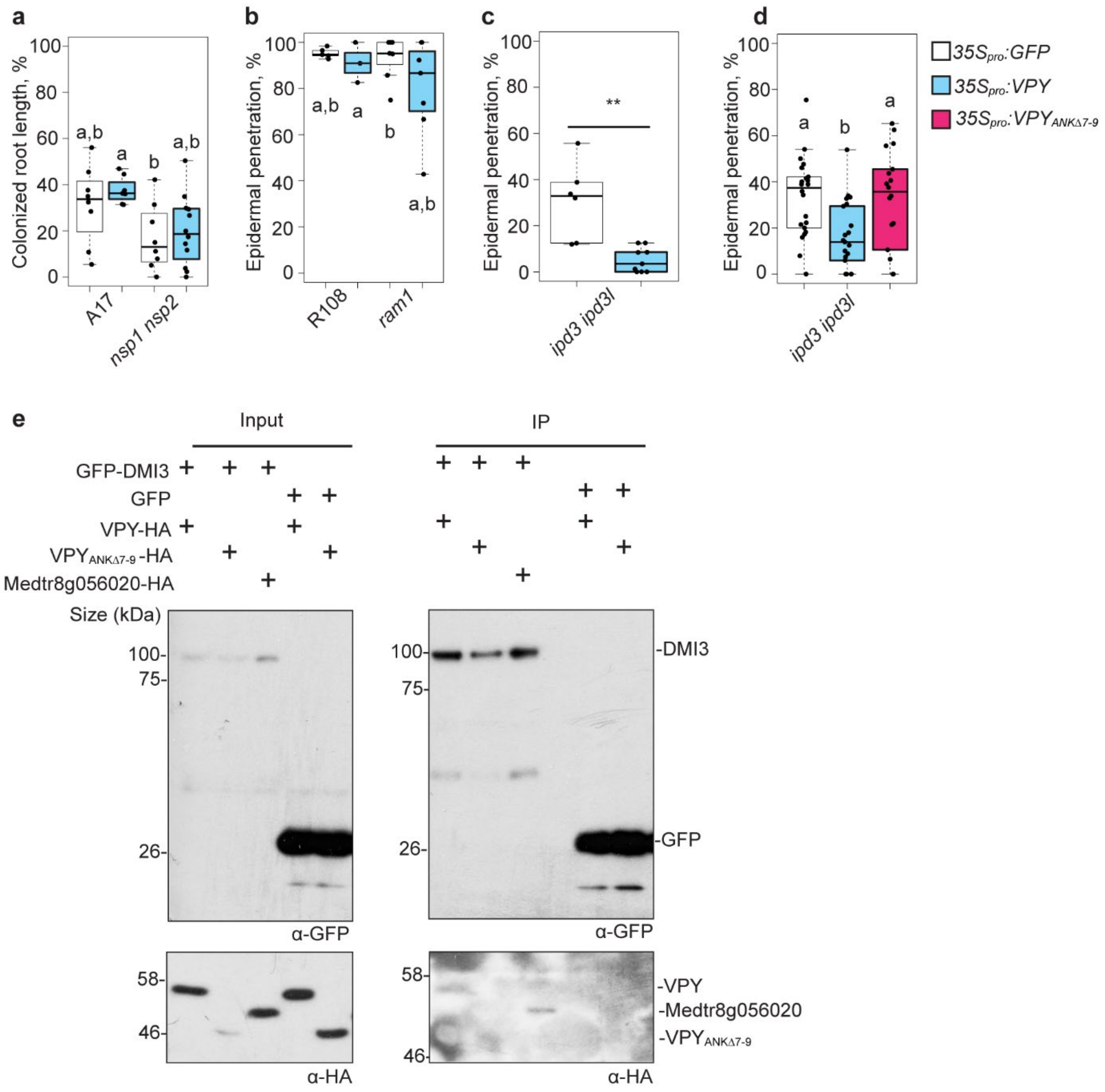

Supplementary Figure 5. Overexpression of VPY does not prevent hyphal entry into *nsp1 nsp2* or *ram1* roots. (a) Ecotype A17 and *nsp1 nsp2*, (b) ecotype R108 and *ram1*, or (c) *ipd3 ipd3l* roots were transformed with *35S$_{pro}$:VPY* or a negative control, *35S$_{pro}$:GFP,* colonized with *D. epigeae,* and harvested four weeks post inoculation. Roots were stained with WGA-AlexaFluor488. (a) Quantification of colonized root length in A17 and *nsp1 nsp2* (n=8 A17 *35S:$_{pro}$GFP*- transformed roots, n=7 in A17 *35S$_{pro}$:VPY*- transformed roots, n=8 in *nsp1 nsp2 35S$_{pro}$:GFP*- transformed roots, n=12 in *nsp1 nsp2 35S$_{pro}$:VPY*- transformed roots). (b) Quantification of epidermal penetration in R108 and *ram1* (n=5 in R108 *35S:$_{pro}$GFP*- transformed roots, n=3 in R108 *35S$_{pro}$:VPY*- transformed roots, n=7 in *ram1 35S$_{pro}$:GFP*-

transformed roots, n=8 in *ram1* $35S_{pro}$*:VPY-* transformed roots). (c) Quantification of epidermal penetration in *ipd3 ipd3l* (n=6 in *35S:* $_{pro}$*GFP-* transformed roots, n=10 in $35S_{pro}$*:VPY-* transformed roots). (d) *ipd3 ipd3l* roots were transformed with either $35S_{pro}$*:VPY* (n=22- transformed roots), $35S_{pro}$*:*$VPY_{ANK\Delta 7\text{-}9}$ (n=18- transformed roots), or $35S_{pro}$*:GFP* (n=22- transformed roots), colonized with *D. epigeae*, and harvested four weeks post inoculation. Epidermal entry was quantified. Different letters indicate statistically significant differences using one-way ANOVA, $p<0.05$, ** signifies $p<0.01$ using a two-tailed Student's t-test. White bars are roots transformed with $35S_{pro}$*:GFP*, blue bars are roots transformed with $35S_{pro}$*:VPY,* and pink bars are roots transformed with $35S_{pro}$*:*$VPY_{ANK\Delta 7\text{-}9}$. In (a) - (d), lines in boxplots represent the median value, box limits represent the upper and lower quartiles, whiskers represent 1.5 times the interquartile range. (e) VPY interacts with DMI3 via ankyrin repeats 7-9. $35S_{pro}$*:VPY-HA,* $35S_{pro}$*:*$VPY_{ANK\Delta 7\text{-}9}$, or $35S_{pro}$*:Medtr8g056020-HA* were co-expressed in *N. benthamiana* with $35S_{pro}$*:GFP-DMI3* or $35S_{pro}$*:GFP*. GFP-DMI3 was immunoprecipitated using anti-GFP magnetic trap beads. VPY-HA and Medtr8g056020-HA co-immunoprecipitated with GFP-DMI3 but $VPY_{ANK\Delta 7\text{-}9}$ was not co-immunoprecipitated with GFP-DMI3 indicating that ankyrin repeats 7-9 are involved in binding. This experiment was repeated once with similar results.

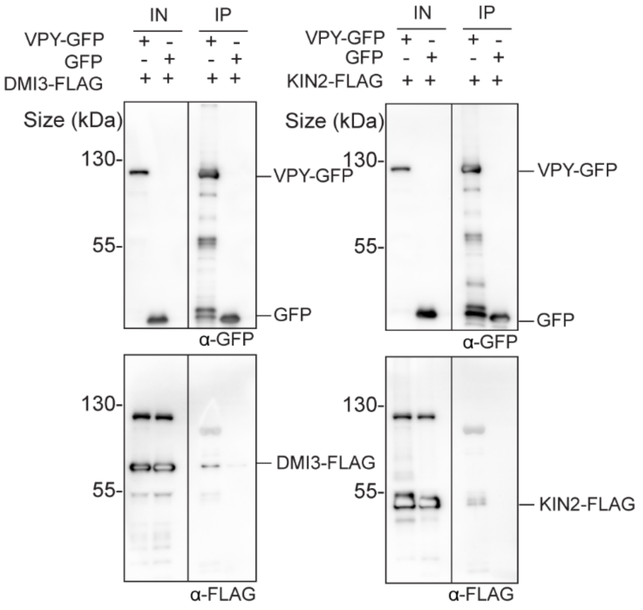

Supplementary Figure 6. VPY-GFP interacts with DMI3-FLAG and KIN2-FLAG. Constructs containing *35S_{pro}:VPY-GFP* and *AtUBQ10p:KIN2-FLAG* or *35S_{pro}:VPY-GFP* and *AtUBQ10p:DMI3-FLAG* were expressed in *N. benthamiana* leaves. VPY-GFP was immunoprecipitated using anti-GFP magnetic trap beads. DMI3-FLAG and KIN2-FLAG co-immunoprecipitated with VPY-GFP. This experiment was repeated once with similar results.

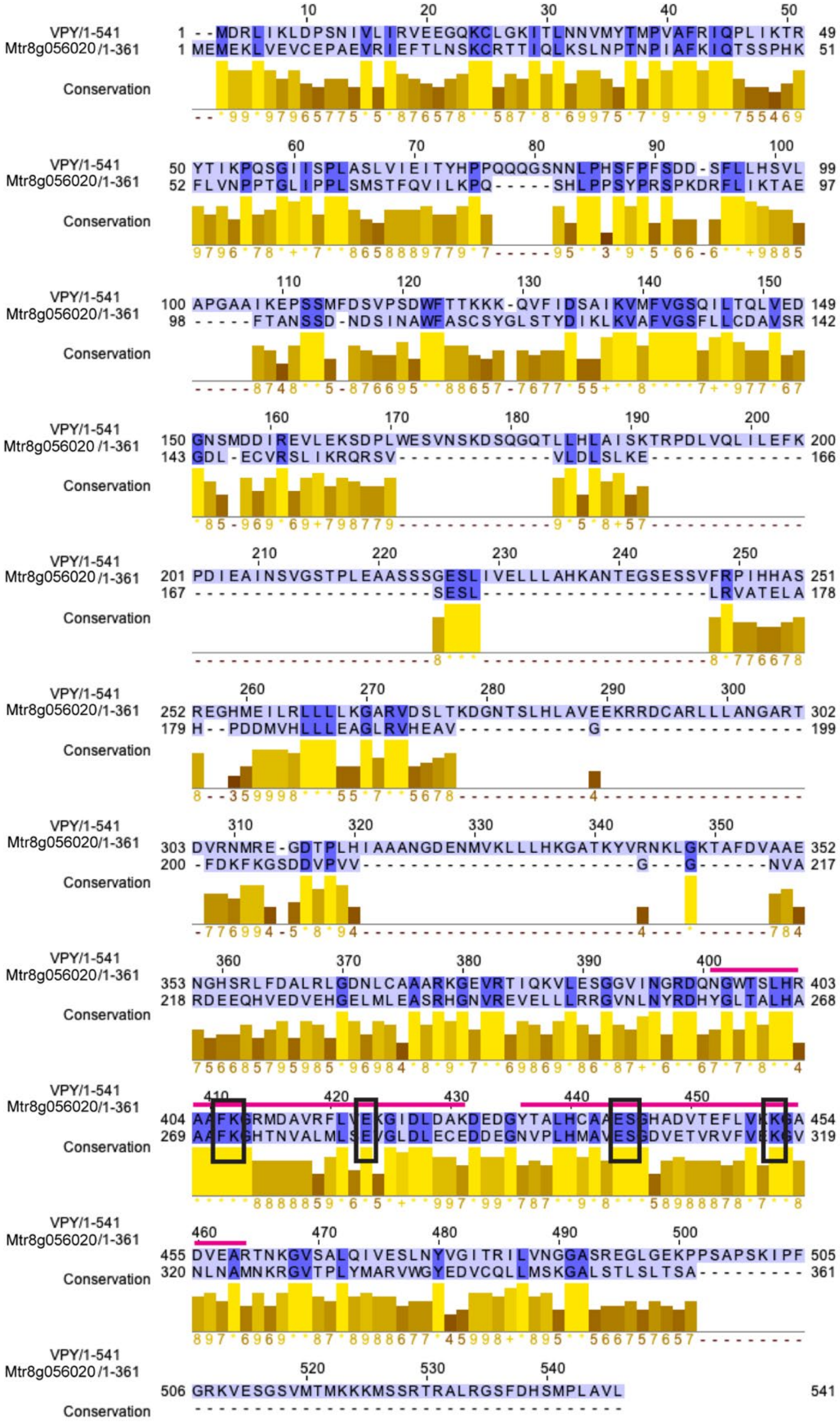

Supplementary Figure 7. Amino acid alignment of VPY and Medtr8g056020. The amino acid sequences of *Medicago truncatula* VPY and Medtr8g056020 were aligned with one another using Clustal Omega and visualized using JalView. Amino acids highlighted in purple are

identical to the consensus sequence, and amino acids highlighted in light purple are similar to the consensus sequence. The level of conservation is indicated as yellow bars below the amino acid sequence. The pink lines indicate ankyrin repeats 7 and 8, black boxes highlight amino acids that are conserved between VPY and Medtr8g056020 which may mediate protein-protein interactions.

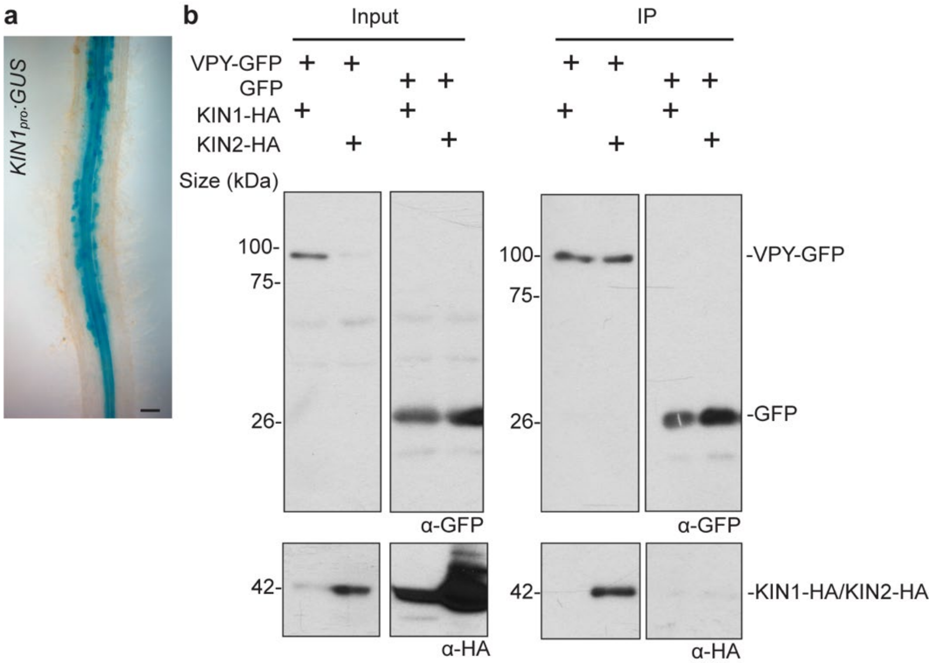

Supplementary Figure 8. VPY does not interact with the sister clade homolog of KIN2, KIN1. (a) Gus staining of a colonized root expressing a KINASE1 promoter GUS fusion. The KINASE1 promoter is active in the cortex of the colonized root. Scale bars are 100 microns. Representative image of five stained transgenic root systems, and eight independent infection units. (b) VPY and KIN1 do not interact as assessed via co-immunoprecipitation. *$35S_{pro}$:VPY-GFP or $35S_{pro}$: GFP* and either *$35S_{pro}$:KIN1-HA* or *$35S_{pro}$:KIN2-HA* were co-expressed in *N. benthamiana* leaves and GFP-tagged proteins were immunoprecipitated using anti-GFP magnetic trap beads. The presence of KIN1-HA and KIN2-HA was assessed via western blot. This experiment was repeated once with similar results.

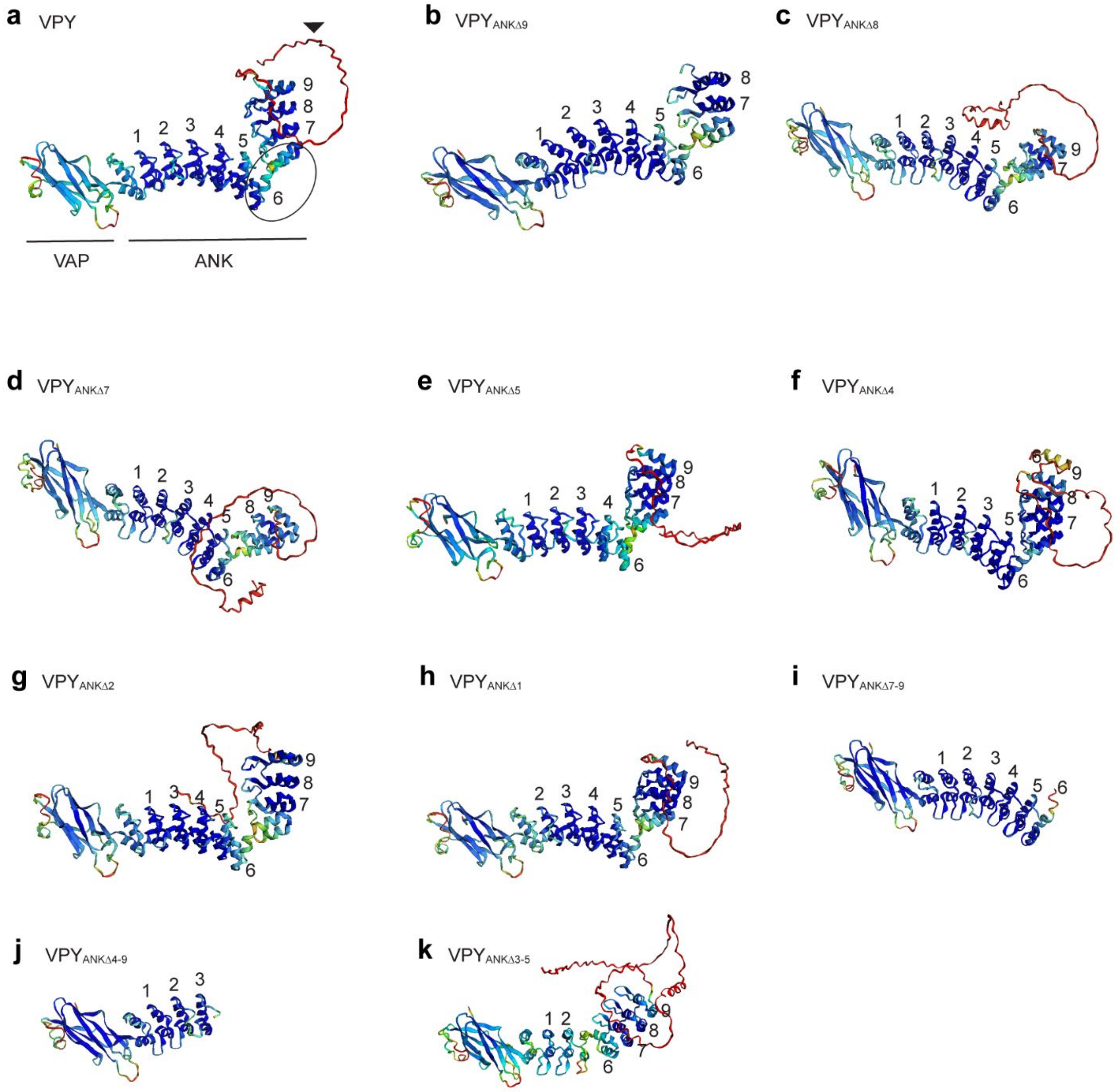

Supplementary Figure 9. AlphaFold predictions of VPY and VPY ankyrin deletions. Ribbon models of (a) VPY, (b) VPY $_{ANK\Delta 9}$, (c) VPY $_{ANK\Delta 8}$, (d) VPY $_{ANK\Delta 7}$, (e) VPY $_{ANK\Delta 5}$, (f) VPY $_{ANK\Delta 4}$, (g) VPY $_{ANK\Delta 2}$, (h) VPY $_{ANK\Delta 1}$, (i) VPY $_{ANK\Delta 7\text{-}9}$, (j) VPY $_{ANK\Delta 4\text{-}9}$, and (k) VPY $_{ANK\Delta 3\text{-}5}$. Numbers indicate ankyrin repeats as outlined in this study, the 'hinge' formed by ankyrin repeat 6 is circled in black, and disordered c-terminal tail is indicated with an arrowhead. Structures are colored based on confidence, with blue being highest confidence prediction, and red being lowest confidence predictions. Note that AlphaFold predicts a less-structured ankyrin repeat between the VAP domain and ankyrin repeat 1.

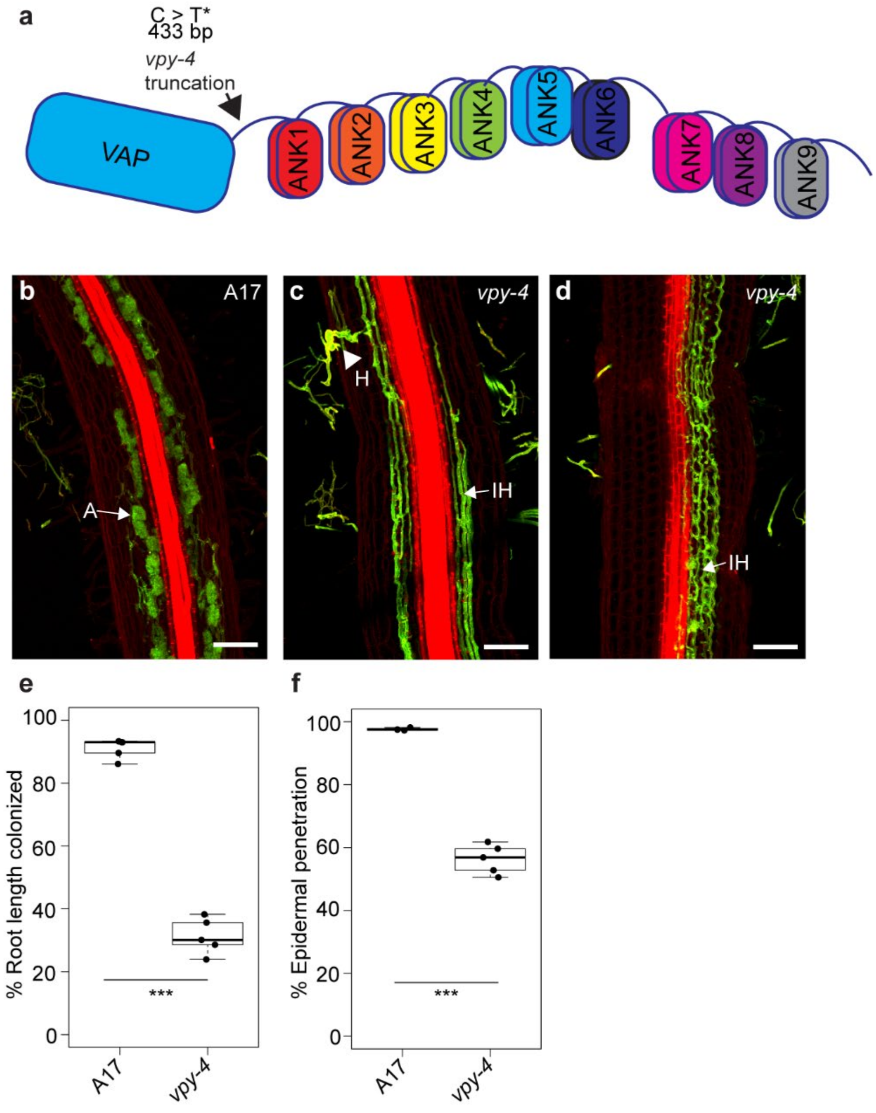

Supplementary Figure 10. *vpy-4* point mutant. *vpy-4* is a new allele. The mutated nucleotide, the site and the corresponding location in the amino acid sequence are indicated in (a). (b) – (d) Confocal images of WT (A17) (b) and *vpy-4* (c - d) roots colonized with *D. epigeae*. Roots were stained with WGA AlexaFluor488 (green) and propidium iodide (red) to visualize fungus and plant cell wall respectively. A= arbuscule, H=hyphopodium, IH=intercellular hyphae. Scale bars are 100 µm. (e) The percentage of root length colonized by *D. epigeae* in the parental WT line A17 and *vpy-4*. (f) The percentage of successful epidermal entry by *D. epigeae* in A17 and *vpy-4*. *** indicates a significance of $p<0.001$ using a two-tailed Student's t-test with WT as compared to *vpy-4*, n =5. Lines in boxplot represent the median value, box limits represent the upper and lower quartiles, whiskers represent 1.5 times the interquartile range.

Supplementary Tables

Supplementary Table 1. Proteins evaluated for interaction with VAPYRIN

Candidate	Gene ID	Rationale	Co-expression coefficient	Interacts (Y/N)
EXO70I	Medtr1g017910	Co-expressor	0.91 (v2)	Y [a]
KINASE2	Medtr4g129010	Co-expressor	0.86 (v3)	Y
KINASE1	Medtr1g102190	Paralog of KINASE2	N/A	N
CYCLIN DEPENDENT KINASE LIKE 1 (CKL1)	Medtr1g098300	Co-expressor	0.83 (v3)	N
CYCLIN DEPENDENT KINASE LIKE 2 (CKL2)	Medtr8g092290	Paralog of CKL1	N/A	N
INTERACTING PARTNER OF DMI3 (IPD3)	Medtr5g026850	Co-expressor	0.81 (v3)	N
Symbiotic Beta Tubulin (TUBB1)	Medtr8g107250	Co-expressor	0.8 (v3)	P[b]
DOES NOT MAKE INFECTIONS 3 (DMI3)	Medtr8g043970	Phenotypic similarity to *vpy*	N/A	Y
DELLA1	Medtr3g065980	Phenotypic similarity to *vpy*	N/A	N

[a] Interaction with EXO70I was shown previously (Zhang et al., 2015).

[b] A possible interaction was observed.

Supplementary Table 2. Construct list

Construct name	Backbone	pDONR4-1	pDONR1-2	pDONR2-3
35S$_{pro}$:GFP (pJL33)	pCAMBIA2300	n/a	n/a	n/a
35S$_{pro}$:VPY	pJL33	n/a	n/a	n/a
35S$_{pro}$:VAP	pJL33	n/a	n/a	n/a
35S$_{pro}$:ANK	pJL33	n/a	n/a	n/a
VPY$_{pro}$:VPY-cpVenus mycRR	pKM43GW mycRR	VPY$_{pro}$	VPY CDS	cpVenus
VPY$_{pro}$:cpVenus-VAP mycRR	pKM43GW mycRR	VPY$_{pro}$	cpVenus	VAP CDS
VPY$_{pro}$:ANK-cpVenus mycRR	pKM43GW mycRR	VPY$_{pro}$	ANK CDS	cpVenus
VPY$_{pro}$:VPY$_{ANK\Delta 9}$-cpVenus mycRR	pKM43GW mycRR	VPY$_{pro}$	VPY$_{ANK\Delta 9}$ CDS	cpVenus
VPY$_{pro}$:VPY$_{ANK\Delta 7\text{-}9}$-cpVenus mycRR	pKM43GW mycRR	VPY$_{pro}$	VPY$_{ANK\Delta 7\text{-}9}$ CDS	cpVenus
VPY$_{pro}$:VPY$_{ANK\Delta 4\text{-}9}$-cpVenus mycRR	pKM43GW mycRR	VPY$_{pro}$	VPY$_{ANK\Delta 4\text{-}9}$ CDS	cpVenus
VPY$_{pro}$:VPY$_{ANK\Delta 3\text{-}5}$-cpVenus mycRR	pKM43GW mycRR	VPY$_{pro}$	VPY$_{ANK\Delta 3\text{-}5}$ CDS	cpVenus
VPY$_{pro}$:VPY$_{ANK\Delta 4}$-cpVenus mycRR	pKM43GW mycRR	VPY$_{pro}$	VPY$_{ANK\Delta 4}$ CDS	cpVenus
VPY$_{pro}$:VPY$_{ANK\Delta 5}$-cpVenus mycRR	pKM43GW mycRR	VPY$_{pro}$	VPY$_{ANK\Delta 5}$ CDS	cpVenus
VPY$_{pro}$:VPY$_{ANK\Delta 1}$-cpVenus mycRR	pKM43GW mycRR	VPY$_{pro}$	VPY$_{ANK\Delta 1}$ CDS	cpVenus
VPY$_{pro}$:VPY$_{ANK\Delta 2}$-cpVenus mycRR	pKM43GW mycRR	VPY$_{pro}$	VPY$_{ANK\Delta 2}$ CDS	cpVenus
VPY$_{pro}$:VPY$_{ANK\Delta 7}$-cpVenus mycRR	pKM43GW mycRR	VPY$_{pro}$	VPY$_{ANK\Delta 7}$ CDS	cpVenus
VPY$_{pro}$:VPY$_{ANK\Delta 8}$-cpVenus mycRR	pKM43GW mycRR	VPY$_{pro}$	VPY$_{ANK\Delta 8}$ CDS	cpVenus
VPY$_{pro}$:VPY-cpVenus RR	pKM43GW RR	VPY$_{pro}$	VPY CDS	cpVenus
VPY$_{pro}$:VPY$_{ANK\Delta 9}$-cpVenus RR	pKM43GW RR	VPY$_{pro}$	VPY$_{ANK\Delta 9}$ CDS	cpVenus
VPY$_{pro}$:VPY$_{ANK\Delta 7\text{-}9}$-cpVenus RR	pKM43GW RR	VPY$_{pro}$	VPY$_{ANK\Delta 7\text{-}9}$ CDS	cpVenus
VPY$_{pro}$:VPY$_{ANK\Delta 4\text{-}9}$-cpVenus RR	pKM43GW RR	VPY$_{pro}$	VPY$_{ANK\Delta 4\text{-}9}$ CDS	cpVenus
VPY$_{pro}$:VPY$_{ANK\Delta 3\text{-}5}$-cpVenus RR	pKM43GW RR	VPY$_{pro}$	VPY$_{ANK\Delta 3\text{-}5}$ CDS	cpVenus
VPY$_{pro}$:VPY$_{ANK\Delta 4}$-cpVenus RR	pKM43GW RR	VPY$_{pro}$	VPY$_{ANK\Delta 4}$ CDS	cpVenus
VPY$_{pro}$:VPY$_{ANK\Delta 5}$-cpVenus RR	pKM43GW RR	VPY$_{pro}$	VPY$_{ANK\Delta 5}$ CDS	cpVenus
VPY$_{pro}$:VPY$_{ANK\Delta 1}$-cpVenus RR	pKM43GW RR	VPY$_{pro}$	VPY$_{ANK\Delta 1}$ CDS	cpVenus
VPY$_{pro}$:VPY$_{ANK\Delta 2}$-cpVenus RR	pKM43GW RR	VPY$_{pro}$	VPY$_{ANK\Delta 2}$ CDS	cpVenus
VPY$_{pro}$:VPY$_{ANK\Delta 7}$-cpVenus RR	pKM43GW RR	VPY$_{pro}$	VPY$_{ANK\Delta 7}$ CDS	cpVenus
VPY$_{pro}$:VPY$_{ANK\Delta 8}$-cpVenus RR	pKM43GW RR	VPY$_{pro}$	VPY$_{ANK\Delta 8}$ CDS	cpVenus
VPY$_{pro}$:VPY-GFP-T35S	pCAMBIA3300	N/A	N/A	N/A
BCP$_{pro}$:mCherry-HDEL	[1]			
BCP$_{pro}$:mCherry-Rab5	[1]			
BCP$_{pro}$:mCherry-Rab7	[1]			
BCP$_{pro}$:mCherry-LC3	[1]			
35S$_{pro}$:GmMan1-mCherry	(Pumplin et al., 2012)			
35S$_{pro}$:mCherry-SYP61	(Pumplin et al., 2012)			
KIN2$_{pro}$:KIN2-GFP	pKM43GW	KIN2$_{pro}$	KIN2 CDS	GFP
KIN2$_{pro}$:GUS	pKM43GW	KIN2 $_{pro}$	GUS	terminator
35S$_{pro}$:VPY-GFP	pKM43GW	35S$_{pro}$	VPY CDS	GFP
35S$_{pro}$:VPY-2XHA	[2]			
35S$_{pro}$:KIN2-HA	pGWB514	N/A	KIN2-CDS	N/A
35S$_{pro}$:KIN1-HA	pGWB514	N/A	KIN1-CDS	N/A
KIN1$_{pro}$:GUS	pKM43GW	KIN1$_{pro}$	GUS	terminator
35S$_{pro}$:GFP	[3]			
35S$_{pro}$:GFP-DMI3	pKM43GW	35S$_{pro}$	GFP	DMI3
35S$_{pro}$:GFP-ΔDELLA1	pKM43GW	35S$_{pro}$	GFP	ΔDELLA1
35S$_{pro}$:TUBB1-GFP	pKM43GW	35S$_{pro}$	TUBB1 CDS	GFP
35S$_{pro}$:VPY$_{ANK\Delta 3\text{-}5}$-HA	pGWB514	N/A	VPY$_{ANK\Delta 3\text{-}5}$ CDS	N/A
35S$_{pro}$:VPY$_{ANK\Delta 7\text{-}9}$-HA	pGWB514	N/A	VPY$_{ANK\Delta 7\text{-}9}$ CDS	N/A
35S$_{pro}$:GOF-DMI3	(Lindsay et al., 2019)			
35S$_{pro}$:Mtr8g056020-GFP	pKM43GW	35S$_{pro}$	Mtr8g056020	GFP
35S$_{pro}$:Mtr8g056020-HA	pGWB514	N/A	Mtr8g056020	N/A
His-MBP-KINASE2	pDEST His-MBP	N/A	KIN2-CDS	N/A
His-MBP-KINASE2$_{K70N}$	pDEST His-MBP	N/A	KIN2$_{K70N}$-CDS	N/A
His-MBP-VPY	pDEST His-MBP	N/A	VPY CDS	N/A

$35S_{pro}$:VPY-cpVenus	pK7m34GW	CaMV35Sp	VPY CDS	cpVenus
$35S_{pro}$:NLS-VPY-cpVenus	pK7m34GW	CaMV35Sp	NLS-VPY	cpVenus
$35S_{pro}$:VPY-NES1-cpVenus	pK7m34GW	CaMV35Sp	VPY-NES1	cpVenus
$35S_{pro}$:VPY-NES2-cpVenus	pK7m34GW	CaMV35Sp	VPY-NES2	cpVenus
$35S_{pro}$:VPY-NES3-cpVenus	pK7m34GW	CaMV35Sp	VPY-NES3	cpVenus
$35S_{pro}$:cpVenus	pK7m34GW	CaMV35Sp	cpVenus	T35S
VPY_{pro}:NLS-VPY-cpVenus RR	pK7m34GW RR	VPY_{pro}	NLS-VPY	cpVenus
VPY_{pro}:VPY-NES1-cpVenus RR	pK7m34GW RR	VPY_{pro}	VPY-NES1	cpVenus
VPY_{pro}:VPY-NES2-cpVenus RR	pK7m34GW RR	VPY_{pro}	VPY-NES2	cpVenus
VPY_{pro}:VPY-NES3-cpVenus RR	pK7m34GW RR	VPY_{pro}	VPY-NES3	cpVenus
VPY_{pro}:NLS-VPY-cpVenus MycRR	pK7m34GW MycRR	VPY_{pro}	NLS-VPY	cpVenus
VPY_{pro}:VPY-NES1-cpVenus MycRR	pK7m34GW MycRR	VPY_{pro}	VPY-NES1	cpVenus
pCAMBIA2300 $35S_{pro}$:VPY-GFP-t35S_AtUBQ10_{pro}:DMI3-FLAG-t35S				
pCAMBIA2300 $35S_{pro}$:VPY-GFP-t35S_AtUBQ10_{pro}:KIN2-FLAG-t35S				
pCAMBIA2300 $35S_{pro}$:GFP-t35S_AtUBQ10_{pro}:DMI3-FLAG-t35S				
pCAMBIA2300 $35S_{pro}$:GFP-t35S_AtUBQ10_{pro}:KIN2-FLAG-t35S				

Supplementary Table 3. Primer list

Primer number	Primer name	Sequence
B2245	VPY full-length overexpression F	GGGGACAAGTTTGTACAAAAAAGCAGGCTTCACCATGGATAGACTCATAAAG
B2470	VPY full-length overexpression R	GGGGACCACTTTGTACAAGAAAGCTGGGTTCTAAAGAACAGCCAAAGGCAT
B2845	VAP overexpression R	GGGGACCACTTTGTACAAGAAAGCTGGGTGGACTTTGATTGCACTATCAA
B2589	ANK overexpression F	GGGGACAAGTTTGTACAAAAAAGCAGGCTTCATGTTTGTTGGTTCACAAATT
B2969	M13 F	TGTAAAACGACGGCCAGT
B2970	M13 R	AGGAAACAGCTATGACCAT
B1460	VPY genotyping F	GAGACTGCAGATGGATAGACTCATAAAGTTAGATCCA
B2398	VPY genotyping R	CATCGGTTCTCGCCCCGTTCGC
B3014	VPY_{pro} attB4-F	**GGGGACAACTTTGTATAGAAAAGTTG**CGGTAAGGGTTACATAAAAGATCG
B3015	VPY_{pro} attB1-R	**GGGGACTGCTTTTTTGTACAAACTTGC**GTTTTTGGTTTAGGGTTTGTATTGA
B3016	VPY attB1-F	**GGGGACAAGTTTGTACAAAAAAGCAGGCTTC**ATGGATAGACTCATAAAGTTAGATCCA
B3017	VPY attB2-R	**GGGGACCACTTTGTACAAGAAAGCTGGGTC**AAGAACAGCCAAAGGCATTGA
B5518	VPY VAP attB2-R	**GGGGACCACTTTGTACAAGAAAGCTGGGTC**CTTTGATTGCACTATCAATGAAAA
B5519	VPY ANK attB1F	**GGGGACAAGTTTGTACAAAAAAGCAGGCTTC**TCATGTTTGTTGGTTCACAA
B6278	$VPY_{ANK\Delta9}$ attB2-R	**GGGGACCACTTTGTACAAGAAAGCTGGGTC**TGCTTCAACATCAGCACCTTT
B6279	$VPY_{ANK\Delta7\text{-}9}$ attB2-R	**GGGGACCACTTTGTACAAGAAAGCTGGGTC**AGCCGCACATAAATTGTCC
B6280	$VPY_{ANK\Delta4\text{-}9}$ attB2-R	**GGGGACCACTTTGTACAAGAAAGCTGGGTC**TGAGTCAACTCTTGCACCTTTG
B6305	$VPY_{ANK\Delta3\text{-}5}$ overlapping F	TAATACAGAAGGATCAGAATCTCTAGGGAA
B6306	$VPY_{ANK\Delta3\text{-}5}$ overlapping R	TTCCCTAGAGATTCTGATCCTTCTGTATTA
B7502	$VPY_{ANK\Delta4}$ overlapping F	TGACTCATTGACAAAAGAAGGTGA
B7503	$VPY_{ANK\Delta4}$ overlapping R	TCACCTTCTTTTGTCAATGAGTCA
B7504	$VPY_{ANK\Delta5}$ overlapping F	TTCGAAACATGAGACTAGGGAAAA
B7505	$VPY_{ANK\Delta5}$ overlapping R	TTTTCCCTAGTCTCATGTTTCGAA
B7498	$VPY_{ANK\Delta1}$ overlapping F	CAAAAGACTCAGTTGGGTCAA

B7499	VPY$_{ANK\Delta 1}$ overlapping R	TTGACCCAACTGAGTCTTTTG
B7500	VPY$_{ANK\Delta 2}$ overlapping F	TTGAAGCTATAAATAGTTCGGTTTTCC
B7501	VPY$_{ANK\Delta 2}$ overlapping R	GGAAAACCGAACTATTTATAGCTTCAA
B7506	VPY$_{ANK\Delta 7}$ overlapping F	TGGAAGGGATCAAGGTTATACAGC
B7507	VPY$_{ANK\Delta 7}$ overlapping R	GCTGTATAACCTTGATCCCTTCCA
B7508	VPY$_{ANK\Delta 8}$ overlapping F	TGCAAAAGATGAAGATAAAGGTGT
B7509	VPY$_{ANK\Delta 8}$ overlapping R	ACACCTTTATCTTCATCTTTTGCA
B6144	cpVenus attB2-F	GGGGACAGCTTTCTTGTACAAAGTGGAAatggacggcggcgtgcag
B6145	cpVenus attB3-R	GGGGACAACTTTGTATAATAAAGTTGCttactcgatgttgtggcgga
B5556	VPY CDS attB2F	GGGGACAGCTTTCTTGTACAAAGTGGAAATGGATAGACTCATAAAGTTAGATCCA
B6026	KIN2$_{pro}$ attb4F	**GGGGACAACTTTGTATAGAAAAGTTG**CTCATTCCGTCTCAAAATATAAGC
B6027	KIN2$_{pro}$ attB1R	**GGGGACTGCTTTTTTGTACAAACTTG**AATTGGTAGTACTATGTTACTATGTTGTTATG
B4998	KIN2 attB1F	**GGGGACAAGTTTGTACAAAAAAGCAGGCTTC**ATGGGATCTTCCTTGAGTTGTT
B5001	KIN2- stop attB2R	GGGGACCACTTTGTACAAGAAAGCTGGGTCttagccaattttcttgtaaggatc
B6028	KIN2-ns attB2R	**GGGGACCACTTTGTACAAGAAAGCTGGGTC**GCCAATTTTCTTGTAAGGATCTTT
B6600	TUBB1 attB1F	**GGGGACAAGTTTGTACAAAAAAGCAGGCTTC**ATGCGTGAGATTCTTCACATTCA
B6601	TUBB1-ns attB2R	**GGGGACCACTTTGTACAAGAAAGCTGGGTC**GCTTTGAGCATCTTCTTCTTCT
B7281	Mtr8g056020 attB1F	**GGGGACAAGTTTGTACAAAAAAGCAGGCTTC** ATGGAGATGGAGAAACTAGTAGAAGT
B7282	Mtr8g056020 attB2R	**GGGGACCACTTTGTACAAGAAAGCTGGGTC** AGCTGAAGTAAGAGAAAGAGTAGACA
B7380	KIN2$_{K70N}$-F	CTACAGATAGCAGTG**AAC**AAACTGAAAGCA
B7381	KIN2$_{K70N}$-R	TGCTTTCAGTTT**GTT**CACTGCTATCTGTAG
B6029	KIN1$_{pro}$ attB4F	**GGGGACAACTTTGTATAGAAAAGTTG**ACAACTACAACAGTACAAAAGGAAGTT

B6030	KIN1$_{pro}$ attB1R	**GGGGACTGCTTTTTTGTACAAACTTG**ATTTCTTTT GTGATAGAATCTCTAACTAACT
B6031	KIN1 attB1F	**GGGGACAAGTTTGTACAAAAAAGCAGGCTTC**AT GCCCTTGAAATGTTTATGTTG
B6032	KIN1-ns attB2R	**GGGGACCACTTTGTACAAGAAAGCTGGGTC**TCTC ATTTTGCGCCTATCATT
B3951	35S$_{pro}$ attB4F	**GGGGACAACTTTGTATAGAAAAGTTG**TGGTG GAGCACGACACTCTC
B3952	35S$_{pro}$ attB1R	**GGGGACTGCTTTTTTGTACAAACTTGC**AGAG ATAGATTTGTAGAGAGAGACTGG
B4139	VAPYRINp-SmaI-F	GGCCCGGGCGGTAAGGGTTACATAAAAGATCG
B3904	T35S-BamHI-R	GGGGATCCGTCACTGGATTTTGGTTTTAGGA
B3189	NLS-attB1-F	GGGGACAAGTTTGTACAAAAAAGCAGGCTTCATGC AGCCTTCTCTTAAACG
B7941	NLS-VPY-F	CCATCTTCTCAACCTGATAGACTCATAAAG
B7942	NLS-VPY-R	CTTTATGAGTCTATCAGGTTGAGAAGATGG
B8159	VPY-ANKnes-attB2-R	GGGGACCACTTTGTACAAGAAAGCTGGGTCCAAAG TCAAAGCTTGCAATCTAGC
B8160	VPY-ANKnes-R	CAAAGTCAAAGCTTGCAATCTAGCATTCAAAAGAA CAGCCAAAGGCATTGA
B8161	VPY-LALnes-attB2-R	GGGGACCACTTTGTACAAGAAAGCTGGGTCAATAT CCAAACCAGCCAATTTC
B8162	VPY-LALnes-R	AATATCCAAACCAGCCAATTTCAAAGCCAAAAGAA CAGCCAAAGGCATTGA
B8163	VPY-TUBnes-attB2-R	GGGGACCACTTTGTACAAGAAAGCTGGGTCCAAAA CCAAATCAATAATTTCTTTACC
B8164	VPY-TUBnes-R	CAAAACCAAATCAATAATTTCTTTACCAATAAGAA CAGCCAAAGGCATTGA
B7939	FLAG-attB2-F	GGGGACAGCTTTCTTGTACAAAGTGGAAGACTACA AGGACGACGATGACAAA
B7940	FLAG-st-attB3-R	GGGGACAACTTTGTATAATAAAGTTGCttaTTTGTCA TCGTCGTCCTTGTAGTC

Supplementary Methods

Detailed methods describing the cloning and assembly of constructs

PCR amplification of gene or promoter fragments

VPY, *KIN1*, *KIN2*, and *TUBB1* promoters and coding regions

To amplify the *VPY*, *KIN1* and *KIN2* promoters, a region spanning 1.5 kb upstream of the ATG start site was designated as the promoter region and amplified using A17 genomic DNA as a template with the following primers: B3014 and B3015 (VPY_{pro}), B6029 and B6030 ($KIN1_{pro}$) and B6026 and B6027 ($KIN2_{pro}$). To amplify the $CaMV35_{pro}$, we amplified the promoter region from pCAMBIA3300 using B3951 and B3952 which contains flanking attB4F and attB1R recombination sites.

KIN1, *KIN2*, *TUBB1*, and *VPY* coding sequences were amplified from A17 *D. epigeae*-colonized root cDNA. The primer combinations for amplifying the coding regions are as follows: *KIN1* - B6031 and B6032, *KIN2* – B4998 and B6028, *TUBB1* – B6600 and B6601, *VPY* – B3016 and B3017.

VPY ankyrin repeat deletion constructs

To clone VPY ankyrin deletion constructs for sub-cellular localization and complementation, we used the pENTR1-2 VPY CDS clone as a template. The following primer sets were used for PCR amplification: B3016 and B6278 for $VPY_{ANK\Delta 9}$, B3016 and B6279 for $VPY_{ANK\Delta 7\text{-}9}$, and B3016 and B6280 for $VPY_{ANK\Delta 3\text{-}9}$. These generate constructs that lack the designated ankyrin repeats and also lack the C-terminal tail region that extends beyond repeat 9.

To clone $VPY_{ANK\Delta 3\text{-}5}$, we generated two PCR fragments using the following two sets of primers: B3016 and B6306 spanning the VAP domain up to the end of ANK2 with B6306 containing a 3' sequence complementary to the beginning of ANK6, and B6305 and B3017 spanning the beginning of ANK6 to ANK9 with B6305 containing a 5' sequence complementary to the end of ANK2. Then, the two PCR products were purified, diluted 1000 times and combined, to perform overlapping PCR using primers B3016 and B3017.

For $VPY_{ANK\Delta 1}$ we amplified two PCR fragments with primers B3016 and B7499 spanning the VAP domain and linker region between the VAP and ANK domain, with B7499 containing a

3’ sequence complementary to the beginning of ANK2, and B7498 and B3017 spanning ANK2 to ANK9, with B7498 containing a 5’ sequence complementary to the linker region. Then, the two PCR products were diluted 1000 times and combined, and overlapping PCR was performed using primers B3016 and B3017.

For VPY$_{ANK\ \Delta 2}$, we amplified two PCR products for overlapping PCR using primer sets B3016 and B7501 which spans the VAP domain up to ANK1 with B7501 containing a 3’ sequence complementary to ANK3, and B7500 and B3017 which spans ANK3 to ANK9, with B7500 containing a 5’ sequence complementary to ANK1. Then, the two PCR products were diluted 1000 times and combined, and overlapping PCR was performed using primers B3016 and B3017.

To clone VPY$_{ANK\Delta 4}$ we generated two PCR fragments using primer sets B3016 and B7503 spanning the VAP domain to the end of ANK3 with a 3’ sequence complementary to the beginning of ANK5, and B7502 and B3017 spanning ANK5 to ANK9, with B7502 containing a 5’ sequence complementary to the beginning of ANK3. Then, the two PCR products were diluted 1000 times and combined, and overlapping PCR was performed using primers B3016 and B3017.

To clone VPY$_{ANK\Delta 5}$, we generated two PCR fragments using primers B3016 and B7505 spanning the VAP domain to the end of ANK4 with B7505 containing a 3’ sequence complementary to the beginning of ANK6, and B7504 and B3017 spanning ANK 6 to ANK9, with B7504 containing a 5’ sequence complementary to the end of ANK4. Then, the two PCR products were diluted 1000 times and combined, and overlapping PCR was performed using primers B3016 and B3017.

For VPY$_{ANK\ \Delta 7}$ we amplified two PCR products using primers B3016 and B7507 spanning the VAP domain up to ANK6 with B7507 containing a 3’ sequence complementary to ANK8, and B7506 and B3017 spanning ANK8 to ANK9 with B7506 containing a 5’ sequence complementary to ANK6. Then, the two PCR products were diluted 1000 times and combined, and overlapping PCR was performed using primers B3016 and B3017.

For VPY $_{ANK\ \Delta 8}$ we amplified two PCR products using B3016 and B7509 spanning the VAP domain up to ANK7 with B7509 containing a 3’ sequence complementary to ANK9, and B7508 and B3017 spanning ANK9, with a 5’ sequence complementary to ANK7. Then, the two PCR products were purified, diluted 1000 times and combined, and overlapping PCR was performed using primers B3016 and B3017.

VAP/MSP and Ankyrin repeat domain constructs

For the individual domain constructs, the *VPY* gene was truncated into two fragments at the nucleotide 405 bp downstream of the ATG of *VPY* coding sequence. The first fragment encodes the VAMP-associated protein (VAP)/major sperm protein domain (*VPY*VAP domain) including amino acids 1 to 135 of *VPY* protein, and the other fragment is carboxyl-terminus containing nine ankyrin repeats (*VPY*ANK domain, amino acids 136 to 541) of *VPY*. The primers used for full-length amplification of the *VPY* ORF were B2245 and B2470. Primers used for truncated *VPY* amplification were B2245 and B2845 (*VPYVAP*), and B2589 and B2470 (*VPYANK*).

Catalytically Inactive form of KINASE2

To clone the catalytically inactive form of KINASE2 in which Lysine 70 is replaced by Asparagine, which blocks ATP binding, we generated two fragments for overlapping PCR using the following primers: B4998 and B7381, and B6028 and B7380. B7381 and B7380 contain point mutations that changes the codon that encodes lysine 70 to an asparagine. Then, the two PCR products were diluted 1000 times and combined, and overlapping PCR was performed using primers B4998 and B6028.

cpVenus

The cpVenus fluorescent protein sequence was amplified B6144 and B6145 using a plasmid template [4]. All primer sequences are in Table S3.

All PCR products were amplified using Phusion HF polymerase (ThermoFisher) and gel purified before recombination into appropriate pDONR vectors.

Generation of pENTR clones

VPY_{pro}, $35S_{pro}$, $KIN1_{pro}$, and $KIN2_{pro}$ PCR products with flanking attB4F and attB1R sites were recombined into pDONR4-1 with BP Clonase (Invitrogen) to obtain pENTR4-1 clones (Table S2).

VPY, $VPY_{ANK\Delta9}$, $VPY_{ANK\Delta7\text{-}9}$, $VPY_{ANK\Delta4\text{-}9}$, $VPY_{ANK\Delta3\text{-}5}$, $VPY_{ANK\Delta4}$, $VPY_{ANK\Delta5}$, $VPY_{ANK\Delta1}$, $VPY_{ANK\Delta2}$, $VPY_{ANK\Delta7}$, $VPY_{ANK\Delta8}$, *ANK, KIN1, KIN2,* and *TUBB1* coding sequence PCR fragments

with flanking attB1F and attB2R sites were recombined into pDONR221 with BP Clonase (Invitrogen) to obtain pENTR1-2 clones (Table S2).

VAP and *cpVenus* coding sequence PCR fragments with flanking attB2F and attB3R sites were recombined into pDONR2-3 with BP Clonase (Invitrogen) to obtain pENTR2-3 clones (Table S2).

Following recombination, all entry clones were transformed into DH5α *E. coli*. Plasmids were verified by sequencing and restriction digest.

Binary vectors for ankyrin deletion complementation analysis

For complementation analysis, the pENTR 4-1 VPY_{pro}, pENTR 1-2 VPY or ankyrin deletions, and pENTR 2-3 cpVenus pENTR clones were recombined into the vector backbone pKM43GW RedRoot containing the DsRed marker [5] using LR Clonase (Invitrogen, Table S2). Clones were propagated in DH5α *E. coli*. Plasmids were transformed into *Arqua1 A. rhizogenes* for hairy root transformation.

Binary vectors for subcellular localization

For subcellular localization, a multisite Gateway reaction was performed with the components listed in Table S2, with pKM43GW MycRedRoot as the vector backbone [6]. For subcellular localization, the following constructs were generated: *VPY_{pro}:VPY-cpVenus mycRR, VPY_{pro}:cpVenus-VAP mycRR, VPY_{pro}:ANK-cpVenus mycRR, VPY_{pro}:$VPY_{ANK\Delta 9}$-cpVenus mycRR, VPY_{pro}:$VPY_{ANK\Delta 7\text{-}9}$-cpVenus mycRR, VPY_{pro}:$VPY_{ANK\Delta 4\text{-}9}$-cpVenus mycRR, VPY_{pro}:$VPY_{ANK\Delta 3\text{-}5}$-cpVenus mycRR, VPY_{pro}:$VPY_{ANK\Delta 4}$-cpVenus mycRR, VPY_{pro}:$VPY_{ANK\Delta 5}$-cpVenus mycRR, VPY_{pro}:$VPY_{ANK\Delta 1}$-cpVenus mycRR, VPY_{pro}:$VPY_{ANK\Delta 2}$-cpVenus mycRR, VPY_{pro}:$VPY_{ANK\Delta 7}$-cpVenus mycRR, VPY_{pro}:$VPY_{ANK\Delta 8}$-cpVenus mycRR.*

For subcellular localization of KIN2, *$KIN2_{pro}$:KIN2-GFP* was generated using a multisite Gateway reaction was performed using pENTR4-1 $KIN2_{pro}$, pENTR1-2 KIN2 CDS, and pENTR2-3 GFP entry clones that were recombined into pKM43GW using LR Clonase (Invitrogen). Clones were propagated in DH5α *E. coli*. Plasmids were transformed into *Arqua1 A. rhizogenes* for hairy root transformation.

Promoter GUS constructs

To clone *KIN1 and KIN2* promoter GUS constructs, a multi-site LR reaction was performed with pKM43GW as the vector backbone, the promoter fragment in pDONR4-1, GUS in pDONR1-2, and a 35S terminator in pDONR2-3. Clones were propagated in DH5α *E. coli*. Plasmids were transformed into *Arqua1 A. rhizogenes* for hairy root transformation.

VPY overexpression constructs

Full-length and truncated *VPY* fragments were amplified from cDNA of *M. truncatula* roots, and subsequently cloned into *pEarleyGate100* to create *ProCaMV35S:VPY*, *ProCaMV35S:VPYVAP* and *ProCaMV35S:VPYANK* constructs. Overexpression constructs of full-length and truncated *VPY* were then amplified using B2969 and B2970, and later inserted into the EcoRI site of *pJL33* [5, 7] to create double constructs with GFP marker, *ProCaMV35S:VPY* + *ProCaMV35S:GFP*, *ProCaMV35S:VPYVAP* + *ProCaMV35S:GFP*, and *ProCaMV35S:VPYANK* + *ProCaMV35S:GFP* constructs. Clones were propagated in DH5α *E. coli*. Plasmids were transformed into *Arqua1 A. rhizogenes* for hairy root transformation.

Constructs for expression in *N. benthamiana*

For co-immunoprecipitation experiments, the $35S_{pro}$*:VPY-GFP,* $35S_{pro}$*:TUBB1-GFP*, and $35S_{pro}$*:Mtr8g056020-GFP* constructs were cloned using a multi-site LR reaction with pKM43GW as the vector backbone, $35S_{pro}$ in pDONR4-1, CDS of interest in pDONR1-2, and GFP in pDONR2-3. The $35S_{pro}$*:GFP-DMI3* and $35S_{pro}$*:GFPΔ DELLA1* constructs were generated using a multi-site LR reaction into pKM43GW destination vector with pENTR 4-1 $35S_{pro}$, pENTR 1-2 GFP, and pENTR 2-3 DMI3 or Δ DELLA1. The $35S_{pro}$*:KIN2-HA,* $35S_{pro}$*:KIN1-HA,* $35S_{pro}$*:*$VPY_{ANK\Delta 3\text{-}5}$*-HA,* $35S_{pro}$*: Mtr8g056020-HA,* and $35S_{pro}$*:*$VPY_{ANK\Delta 7\text{-}9}$*-HA* constructs were constructed using a single site LR reaction with pGWB514 as a vector backbone and the coding sequence of interest in pDONR1-2. Clones were propagated in DH5α *E. coli*. Plasmids for expression in *N. benthamiana* were transformed into GV3101 *Agrobacterium tumefaciens*.

Constructs for recombinant protein expression in *E.coli*

For recombinant protein production in *E. coli*, VPY, KIN2, and $KIN2_{K70N}$ pENTR1-2 clones were cloned into the His-MBP Destination vector [8]. Clones were propagated in DH5α *E. coli* and then extracted plasmids were transformed into Rosetta BL (DE1) *E. coli* for recombinant protein expression.

VPY-GFP construct in pCambia3300

First, pK7m34GW *VPY_{pro}:VPY-GFP* was created using multisite Gateway.
Second, the *VPY_{pro}:VPY-GFP-T35S* expression cassette was amplified using VPYp-SmaI-F(B4139) and T35S-BamHI-R(B3904). Third, SmaI-VPY_{pro}:VPY-GFP-T35S-BamHI and pCAMBIA3300 were digested by SmaI and BamHI and used in ligation reaction using T4 DNA Ligase (NEB). pCAMBIA3300 *VPY_{pro}:VPY-GFP-T35S* was transformed into *A. tumefaciens* AGL1. This plasmid and strain was used in *Medicago truncatula* R108 stable transformation.

Nuclear localization signal (NLS) and nuclear exclusion signal (NES) fusions of VPY

To create translational fusion of *VPY* with nuclear localization signal (*NLS*) the *VPY* coding sequence was amplified using B7941 and B3017 primers and pENTR1-2 VPY CDS clone as a template and *NLS* sequence was amplified using B3189 and B7942 primers and pENTR1-2 NLS-GFP-GUS clone [9] as a template. Obtained PCR fragments were used as templates in overlapping PCR with B3189 and B3017 primers to create *NLS-VPY* sequence. Obtained PCR fragment with flanking *att*B sites was recombined in with pDONR221 to obtain pENTR1-2 NLS-VPY CDS clone using Gateway™ BP Clonase™ II Enzyme mix.
To create translational fusion of *VPY* with nuclear exclusion signals (*NES*) the *VPY* coding sequence was amplified using pENTR1-2 VPY CDS clone as a template and primers B3016 and B8160 (*NES1*), B8162 (*NES2*) or B8164 (*NES3*). Obtained PCR fragments were used in following PCR with primers B3016 and B8159 (*NES1*), B8161(*NES2*) or B8163(*NES3*) to complete the fusions. *NES1* (LNARLQALTL) is from the mammalian ankyrin-repeat protein ANKLE 1 [10]. *NES2* (LALKLAGLDI) is described in Wu and Spalding 2007 [11]. *NES3* (IGKEIIDLVL) is from a tubulin gene [12]. Obtained PCR fragments with flanking *att*B sites were recombined in with pDONR221 to obtain pENTR1-2 *VPY-NES1*, pENTR1-2 *VPY-NES2* or pENTR1-2 *VPY-NES3* clones using Gateway™ BP Clonase™ II Enzyme mix.

To test the subcellular location of NLS or NES fusions of VPY in *N. benthamiana* leaves the pENTR1-2 clones were used in three fragment recombination with pENTR4-1 *CaMV35Sp*, pENTR2-3 *cpVenus* and pK7m34GW destination vector [13, 14] using Gateway™ LR Clonase™ II Enzyme mix to create expression vectors.
To test the subcellular location of NLS or NES fusions of VPY in *M. truncatula* roots the pENTR1-2 clones were used in three fragment recombination with pENTR4-1 *VPYp*, pENTR2-3 *cpVenus* and pK7m34GW MycRR destination vector. For *vpy* mutant complementation assay using NLS or NES fusions of VPY in *M. truncatula* roots the pENTR1-2 clones were used in three fragment recombination with pENTR4-1 *VPYp*, pENTR2-3 *cpVenus* and pK7m34GW RR destination vector.

VPY-GFP interaction with DMI3-FLAG and KIN2-FLAG

FLAG-tag was created in PCR with B7939 and 7940 primers. Obtained PCR fragment with flanking *att*B sites was recombined in with pDONR P2RP3 to obtain pENTR2-3 FLAG clone using Gateway™ BP Clonase™ II Enzyme mix.
To create *CaMV35Sp:VPY-GFP* expression clone for co-immunoprecipitation from *N. benthamiana* leaf tissue the pENTR1-2 VPY CDS clone was used in three fragment recombination with pENTR4-1 *CaMV35Sp*, pENTR2-3 *GFP* and pK7m34GW destination vector using Gateway™ LR Clonase™ II Enzyme mix. To create *AtUBQ10p:DMI3-FLAG* and *AtUBQ10p:KIN2-FLAG* expression clones the pENTR1-2 DMI3 CDS or pENTR1-2 KIN2 CDS clones were used in three fragment recombination with pENTR4-1 *AtUBQ10p*, pENTR2-3 *FLAG* and pK7m34GW destination vector using Gateway™ LR Clonase™ II Enzyme mix.

For all constructs, the correct plasmid sequences were confirmed via restriction digest and sequencing. The plasmids used during for this study are listed in Table S2. The primers used to generate the plasmids are listed in Table S3.

References

1. Ivanov, S. & Harrison, M.J. A set of fluorescent protein-based markers expressed from constitutive and arbuscular mycorrhiza-inducible promoters to label organelles,

membranes and cytoskeletal elements in Medicago truncatula. *Plant J* **80**, 1151-1163 (2014).

2. Zhang, X., Pumplin, N., Ivanov, S. & Harrison, M.J. EXO70I Is Required for Development of a Sub-domain of the Periarbuscular Membrane during Arbuscular Mycorrhizal Symbiosis. *Curr Biol* **25**, 2189-2195 (2015).
3. Floss, D.S., Levy, J.G., Levesque-Tremblay, V., Pumplin, N. & Harrison, M.J. DELLA proteins regulate arbuscule formation in arbuscular mycorrhizal symbiosis. *Proc Natl Acad Sci U S A* **110**, E5025-5034 (2013).
4. Mukherjee, P. et al. Live Imaging of Inorganic Phosphate in Plants with Cellular and Subcellular Resolution. *Plant Physiol.* **167**, 628-638 (2015).
5. Floss, D.S., Levy, J.G., Levesque-Tremblay, V., Pumplin, N. & Harrison, M.J. DELLA proteins regulate arbuscule formation in arbuscular mycorrhizal symbiosis. *Proceedings of the National Academy of Sciences* **110**, doi:10.1073/pnas.1308973110 (2013).
6. Ivanov, S. & Harrison, M.J. Accumulation of phosphoinositides in distinct regions of the periarbuscular membrane. *New Phytol.* **221**, 2213-2227 (2019).
7. Chiu, W. et al. Engineered GFP as a vital reporter in plants. *Current Biology* **6**, 325-330 (1996).
8. Nallamsetty, S., Austin, B.P., Penrose, K.J. & Waugh, D.S. Gateway vectors for the production of combinatorially-tagged His(6)-MBP fusion proteins in the cytoplasm and periplasm of Escherichia Coli. *Protein Science* **14**, 2964-2971 (2005).
9. Ivanov, S. & Harrison, M.J. A set of fluorescent protein-based markers expressed from constitutive and arbuscular mycorrhiza-inducible promoters to label organelles, membranes and cytoskeletal elements in Medicago truncatula. *Plant J.* **80**, 1151-1163 (2014).
10. Zlopasa, L., Brachner, A. & Foisner, R. Nucleo-cytoplasmic shuttling of the endonuclease ankyrin repeats and LEM domain-containing protein 1 (Ankle1) is mediated by canonical nuclear export- and nuclear import signals. *BMC Cell Biol.* **17** (2016).
11. Wu, G. & Spalding, E.P. Separate functions for nuclear and cytoplasmic cryptochrome 1 during photomorphogenesis of Arabidopsis seedlings. *Proc. Natl. Acad. Sci. USA* **104**, 18813-18818 (2007).
12. Schwarzerova, K. et al. Tubulin is actively exported from the nucleus through the Exportin1/CRM1 pathway. *Scientific Reports* **9** (2019).
13. Karimi, M., Bleys, A., Vanderhaeghen, R. & Hilson, P. Building blocks for plant gene assembly. *Plant Physiol.* **145**, 1183-1191 (2007).
14. Karimi, M., Depicker, A. & Hilson, P. Recombinational cloning with plant gateway vectors. *Plant Physiol.* **145**, 1144-1154 (2007).
