# Supplementary material for: Distinct ankyrin repeat subdomains control VAPYRIN locations and intracellular accommodation functions during arbuscular mycorrhizal symbiosis
Source: Nat Commun. 2022 Sep 5;13:5228. doi: 10.1038/s41467-022-32124-3 (PMC9445082; doi:10.1038/s41467-022-32124-3)
Supplement: Supplementary file 3 — Reporting Summary [file 41467_2022_32124_MOESM3_ESM.pdf]

Corresponding author(s): Maria J Harrison

Last updated by author(s): 7/11/2022

Reporting Summary

Nature Portfolio wishes to improve the reproducibility of the work that we publish. This form provides structure for consistency and transparency in reporting. For further information on Nature Portfolio policies, see our Editorial Policies and the Editorial Policy Checklist.

Statistics

For all statistical analyses, confirm that the following items are present in the figure legend, table legend, main text, or Methods section.

n/a	Confirmed	
☐	☒	The exact sample size (*n*) for each experimental group/condition, given as a discrete number and unit of measurement
☐	☒	A statement on whether measurements were taken from distinct samples or whether the same sample was measured repeatedly
☐	☒	The statistical test(s) used AND whether they are one- or two-sided<br>*Only common tests should be described solely by name; describe more complex techniques in the Methods section.*
☒	☐	A description of all covariates tested
☒	☐	A description of any assumptions or corrections, such as tests of normality and adjustment for multiple comparisons
☒	☐	A full description of the statistical parameters including central tendency (e.g. means) or other basic estimates (e.g. regression coefficient) AND variation (e.g. standard deviation) or associated estimates of uncertainty (e.g. confidence intervals)
☐	☒	For null hypothesis testing, the test statistic (e.g. *F*, *t*, *r*) with confidence intervals, effect sizes, degrees of freedom and *P* value noted<br>*Give P values as exact values whenever suitable.*
☒	☐	For Bayesian analysis, information on the choice of priors and Markov chain Monte Carlo settings
☒	☐	For hierarchical and complex designs, identification of the appropriate level for tests and full reporting of outcomes
☒	☐	Estimates of effect sizes (e.g. Cohen's *d*, Pearson's *r*), indicating how they were calculated

Our web collection on statistics for biologists contains articles on many of the points above.

Software and code

Policy information about availability of computer code

Data collection	Confocal images were collected using the Leica LAS X software.
Data analysis	Data analysis was conducted using R software (RStudio Version 1.4.1106).<br>Colab Fold was used to generate protein structure predictions. This open-source software combines use of AlphaFold2 and MMseqs2 to accelerate prediction of protein structures. It is available as a Google Colab notebook.<br>Fiji was used to process confocal images. The transect analysis in Supplementary Figure 1 was conducted using Leica LAS X.

For manuscripts utilizing custom algorithms or software that are central to the research but not yet described in published literature, software must be made available to editors and reviewers. We strongly encourage code deposition in a community repository (e.g. GitHub). See the Nature Portfolio guidelines for submitting code & software for further information.

Data

Policy information about availability of data

All manuscripts must include a data availability statement. This statement should provide the following information, where applicable:

- Accession codes, unique identifiers, or web links for publicly available datasets
- A description of any restrictions on data availability
- For clinical datasets or third party data, please ensure that the statement adheres to our policy

The plant genetic materials and constructs used in this research will be made freely available on request (contact, mjh78@cornell.edu).

Human research participants

Policy information about studies involving human research participants and Sex and Gender in Research.

Reporting on sex and gender	N/A
Population characteristics	*Describe the covariate-relevant population characteristics of the human research participants (e.g. age, genotypic information, past and current diagnosis and treatment categories). If you filled out the behavioural & social sciences study design questions and have nothing to add here, write "See above."*
Recruitment	*Describe how participants were recruited. Outline any potential self-selection bias or other biases that may be present and how these are likely to impact results.*
Ethics oversight	*Identify the organization(s) that approved the study protocol.*

Note that full information on the approval of the study protocol must also be provided in the manuscript.

Field-specific reporting

Please select the one below that is the best fit for your research. If you are not sure, read the appropriate sections before making your selection.

☒ Life sciences ☐ Behavioural & social sciences ☐ Ecological, evolutionary & environmental sciences

For a reference copy of the document with all sections, see nature.com/documents/nr-reporting-summary-flat.pdf

Life sciences study design

All studies must disclose on these points even when the disclosure is negative.

Sample size	Sample size was determined based on data collection and analysis standards in our field. Sample sizes are reported in the manuscript
Data exclusions	In experiments involving colonization with AM fungi and root transformations, data were excluded only when there was no presence of AM fungi detected, or when root transformations were unsuccessful.
Replication	To ensure reproducibility of findings, experiments were performed with many replicates, as outlined in the manuscript. Experiments were repeated whenever possible. Co-immunprecipitation experiments were repeated several times, with independent biological material used to ensure interactions were consistently observed.
Randomization	Plants subjected to different treatments (eg., inoculation or mock-inoculation) were randomly selected.
Blinding	Blinding was not possible for this study because of personnel constraints. Most phenotypes observed in this study have very obvious differences, but for any subtler effects, we have clearly outlined how we have collected data to minimize bias.

Reporting for specific materials, systems and methods

We require information from authors about some types of materials, experimental systems and methods used in many studies. Here, indicate whether each material, system or method listed is relevant to your study. If you are not sure if a list item applies to your research, read the appropriate section before selecting a response.

Materials & experimental systems

n/a	Involved in the study
☐	☒ Antibodies
☒	☐ Eukaryotic cell lines
☒	☐ Palaeontology and archaeology
☒	☐ Animals and other organisms
☒	☐ Clinical data
☒	☐ Dual use research of concern

Methods

n/a	Involved in the study
☒	☐ ChIP-seq
☒	☐ Flow cytometry
☒	☐ MRI-based neuroimaging

Antibodies

Antibodies used

anti-GFP (Roche, catalog number 11814460001, clones 7.1 and 13.1)
anti-HA (Sigma-Aldrich, catalog number H3663, clone HA-7)
anti-FLAG (Sigma-Aldrich, catalog number F3165, clone M2)

Validation

The GFP, HA, and FLAG antibodies are commercial antibodies that have been routinely demonstrated to work for N. benthamiana co-immunoprecpitation assays. The GFP and HA antibodies have been used in previous publications from our lab:
https://doi.org/10.1016/j.cub.2017.03.003
https://doi.org/10.1016/j.cub.2015.06.075
The FLAG antibody has been used in over 100 publications with N. benthamiana protein studies, including:
https://doi.org/10.1242/dev.048199
https://doi.org/10.1073/pnas.1807297116
